# Identification, expression and functional characterization of M_4_L, a muscarinic acetylcholine M_4_ receptor splice variant

**DOI:** 10.1371/journal.pone.0188330

**Published:** 2017-12-06

**Authors:** Douglas A. Schober, Carrie H. Croy, Cara L. Ruble, Ran Tao, Christian C. Felder

**Affiliations:** 1 Neuroscience, Lilly Research Laboratories, Lilly Corporate Center, Eli Lilly and Company, Indianapolis, Indiana, United States of America; 2 Lieber Institute for Brain Development, Baltimore, Maryland, United States of America; International Centre for Genetic Engineering and Biotechnology, ITALY

## Abstract

Rodent genomic alignment sequences support a 2-exon model for muscarinic M4 receptor. Using this model a novel N-terminal extension was discovered in the human muscarinic acetylcholine M_4_ receptor. An open reading frame was discovered in the human, mouse and rat with a common ATG (methionine start codon) that extended the N-terminus of the muscarinic acetylcholine M_4_ receptor subtype by 155 amino acids resulting in a longer variant. Transcriptional evidence for this splice variant was confirmed by RNA-Seq and RT-PCR experiments performed from human donor brain prefrontal cortices. We detected a human upstream exon indicating the translation of the mature longer M_4_ receptor transcript. The predicted size for the longer two-exon M_4_ receptor splice variant with the additional 155 amino acid N-terminal extension, designated M_4_L is 69.7 kDa compared to the 53 kDa canonical single exon M_4_ receptor (M_4_S). Western blot analysis from a mammalian overexpression system, and saturation radioligand binding with [^3^H]-NMS (N-methyl-scopolamine) demonstrated the expression of this new splice variant. Comparative pharmacological characterization between the M_4_L and M_4_S receptors revealed that both the orthosteric and allosteric binding sites for both receptors were very similar despite the addition of an N-terminal extension.

## Introduction

The endogenous neurotransmitter, acetylcholine, binds to both nicotine-sensitive ion channels and muscarinic-sensitive GPCRs (G-protein coupled receptors). The muscarinic family contains 5 known Class A, membrane protein receptor subtypes (M_1_-M_5_) that originate from distinct genes. The M_4_ receptor is highly expressed in the striatum, cortex, and hippocampus, areas involved in mood, cognition, and drug seeking behaviors. However, relatively little is known about the physiological function of the M_4_ receptor as selective pharmacological tools have only recently been developed [1]. Selective M_4_ positive allosteric modulators have shown efficacy similar to both typical and atypical antipsychotics in animal models predictive of schizophrenia [2, 3]. The M_4_ receptor plays an inhibitory role on presynaptic terminals and regulates neurotransmitter release in both an autoreceptor role (acetylcholine) and hetero-receptor role (e.g. dopamine, GABA, serotonin) depending on brain region localization [4]. To date no mouse, rat or human splice variants of the muscarinic receptors that generate novel proteins have been characterized.

Many GPCRs were thought to originate from single exon genes. The recognition of introns within the coding regions of GPCRs has increased gradually from 10% [5] to 52% [6]. Subject to conditional, temporal, and cell-type regulation, alternative splicing can generate structurally similar proteins with functionally identical or significantly different properties [7] effecting signaling and/or pharmacology [8]. Alternatively spliced GPCR isoforms can differ in their abilities to undergo post-translational modification and/or to interact with accessory proteins which also can greatly influence their biological activity [6].

The extracellular N-terminal extension of M_4_ is short (31 amino acids) compared to other neurotransmitter receptors which typically have long N-terminal segments [9]. The curated transcript sequence in GenBank (NM_00741) transcribes a 1.8 kb product, and the protein sequences (NP_000732) translates into a 53 kDa protein. However published work such as Buchli et al which show experimental northern blot data estimating an M_4_ receptor transcript size of 4.8 kb and a Western blot reactive-protein estimated at 70 kDa [10]. These size discrepancies led us to explore the M_4_ receptor for transcriptional diversity. Expressed Sequence Tags (ESTs) analyses were used to identify additional exons, and comparative genomics analyses were used to identify putative 5’extensions of the open reading frame (ORF). These analyses when performed on rodent and human genomic databases revealed an alternative M_4_ receptor transcript that would increase the extracellular N-terminus by 155 amino acids. Human neuronal RNA-seq data was then interrogated and confirmed the existence of the splice junctions, and the mature mRNA was experimentally confirmed by RT-PCR. Further experiments were performed to confirm and characterize the longer M_4_-receptor variant (M_4_L) protein. Overall the binding and functional pharmacology studies characterized and compared the human muscarinic M_4_L receptor splice variant that encodes an additional 155 amino acids on the N-terminus to that of the canonical single exon M_4_ receptor protein in an overexpressed cellular model.

## Materials and methods

### Human tissues, RNA extraction and quality assessment, RNA-seq library construction, RNA sequence mapping

Human tissues samples used in this study were part of an early-stage research consortium BrainSEQ^™^ with the Lieber Institute of Brain Development (LIBD), with the goal of expanding knowledge around the genetic contribution to brain disorders. The LIBD postmortem human brain collection contains samples acquired through an informed consent from relatives of the deceased for 751 postmortem human brain samples through the Office of the Chief Medical Examiner of the State of Maryland. Additionally, the collection has 1,213 postmortem human brain tissue samples acquired via material transfer agreements, including those from the National Institute of Mental Health (NIMH), the Eunice Kennedy Shriver National Institute of Child Health and Development (NICHD) Brain Bank, the Stanley Medical Research Institute, and The Johns Hopkins University. All brain donations were obtained by verbal, witnessed informed consent with the next-of-kin (protocol #90-M-0142 approved by the NIMH/NIH Institutional Review Board). RNA-Seq datasets from the dorsal lateral prefrontal cortex (DL-PFC) of 211 donors contained an average of 114 million reads per sample, with an average of 81% mapping rate to the reference genome were previously described in Ruble *et al* [11]. Genomic DNA comparisons were done with ACT: the Artemis Comparison Tool [12]. Sim4 was used for aligning mRNA and EST sequences [13]. Open Reading Frame analysis was done using Sequencher^®^ version 5.1 sequence analysis software, Gene Codes Corporation, Ann Arbor, MI USA http://www.genecodes.com. The EMBOSS suite was used for sequence extraction and calculation of molecular weights [14]. Multiple alignments were created using MUSCLE [15] and viewed using GeneDoc [16]. RNA-seq reads were aligned to the human genome reference with GSNAP [17] and visualized using OmicSoft^®^ ArrayStudio^®^ software, version 10.

### PCR and sequence confirmation

To verify the splicing variants of M_4_ in human brain, we performed exon-to-exon PCR using 3 brain total RNA with M_4_ gene specific sense primers binding at exon 1 using SMART RACE cDNA Amplification Kit (Clonetech) and Advantage 2 PCR Kit (Clonetech). The human brain total RNA was reverse-transcribed to cDNA by MMLV reverse transcriptase (Clontech) according to the manufacturer’s protocol. Based on RNA sequencing, we designed primer pairs to verify the M_4_ splice variants using Platinum TaqDNA polymerase (Invitrogen). The control primer set to detect the canonical M_4_ transcript was TCCCACAATCGCTATGAGACG (forward) and CACCACAAACTGCCAGAACAAG (reverse). Junction primers were designed to bridge between the short and long transcripts (GTCCGTCCCGCCGTCTGTCT (forward) and CGTTGCTCACCACGTAGTCC (reverse)). The PCR conditions were 94°C for 3 min, 35 cycles of 94°C for 30 sec, 60°C for 30 sec, 72°C for 1 min, and 72°C for 10 min after the last cycle. The PCR products were cloned into E. coli by PCR-TOPO 4.0 vectors (InvitrogenTM) and sequenced [18]. All PCR results were confirmed in separate PCR assays and Sanger sequencing.

### Western blot assays

Protein was extracted from cell pellets using RIPA buffer and protease inhibitors and soluble protein lysate from this preparation was quantified using Coomassie Plus Protein Assay (Thermo-Fisher). Lysates were normalized by total protein and 10μg loaded on a 4–20% Tris-Glycine gradient gel (Novex, Life Technologies). Samples were transferred onto PVDF membranes, membranes were blocked with 5% nonfat milk phosphate-buffered saline with 0.1% Tween-20 (PBS-T) (Sigma-Aldrich, P9416) for 1h at room temperature prior to antibody incubation. The muscarinic M_4_ receptor was N-terminal tagged with c-MYC so that we could use a rabbit primary antibody which recognized myc epitope (Millipore, 05–724, 1:1000 in 5% milk PBS-T) In addition, a beta actin loading control mouse monoclonal antibody (ThermoFisher, MA5-15739, 1:1000 in 5% milk PBS-T was used to normalize the Western blot. Both antibodies were incubated overnight at 4°C. Blots were washed in PBS-T then probed with mouse and rabbit IRDye secondary antibodies (Li-COR, 1:15,000, 5% milk. PBS-T) and visualized by the Li-COR Odyssey^®^ Imaging system.

### Immunofluorescence

Immunofluorescence analysis of HEK293T (ATCC, CRL-3216) transiently expressing M_4_ receptor were carried out by labelling the constructs N-terminal myc-epitope. Specifically, cells were fixed with 4% paraformaldehyde, permeabilized with 0.25% Triton X-100, blocked in 5% normal goat-serum, incubated with anti-myc 1:1000 (anti-myc, clone A46, Millipore 05–724), and detected via the Alexa Fluor 488-conjugated goat anti-mouse IgG (1:500, Jackson Immunologics) secondary antibody. Hoerst stain (blue) was used as the nuclear counterstain.

### [^3^H]-NMS binding assays

[^3^H]-NMS (PerkinElmer, NET636001MC) saturation binding assays were performed in HEPES buffer [20mM HEPES (Sigma-Aldrich, H0887), 100mM NaCl (S5150), 10mM MgCl_2_ (M1028), pH 7.4]. Equilibrium binding was achieved by incubating 3–5μg of muscarinic-containing membranes (human M_4_S or M_4_L transiently expressed in HEK293T cells) and various concentrations of [^3^H]-NMS (0.01-2nM) for 2h at 25°C. Bound [^3^H]-NMS (fmol/mg membrane) vs. NMS concentration (nM) was fit to a one-site specific binding model using Prism 6.7 (GraphPad Software, Inc.) and was used to calculate B_max_ and K_d_ values. [^3^H]-NMS (~0.35nM) displacement assays were performed for 2h at 25°C in HEPES buffer with membranes (5–10μg) containing either M_4_S or M_4_L in the presence of varying concentrations (0.1-10mM) of a M_4_-PAM (positive allosteric modulator), LY2033298 (Lilly) and acetylcholine (Ach) (Sigma-Aldrich, A6625. All reactions were stopped by rapid filtration on a TOMTEC 96-well cell harvester. Non-specific binding was determined using atropine (10μM) (Sigma-Aldrich, A0132) for both the saturation and displacement binding assays. Radioactivity retained on the filter-mat was counted on a Wallac 1205 Beta-plate scintillation spectrophotometry. The specific binding vs. concentration fit to a one-site K_i_ model using GraphPad Prism 6.7 using established [^3^H]-NMS K_d_ values.

### GTP-γ-[^35^S] binding assays

The level of G protein activation was measured by the amount of non-hydrolyzable GTP-γ-[^35^S] bound to Gαi- subunit. The GTP-γ-[^35^S] (PerkinElmer, NEG030X001MC) binding was determined using a WGA SPA-bead technique. GTP-γ-[^35^S] binding was produced by incubating 50μg of membranes expressing the human M_4_S or M_4_L variants in assay buffer (20mM HEPES, 100mM NaCl, 0.2μM EDTA, 1μM GDP and 10mM MgCl_2_, pH 7.4), 500pM GTP- γ -[^35^S], and varying concentrations of orthosteric muscarinic agonists (acetylcholine, oxotremorine-M and McN-A-343 and pilocarpine). Binding proceeded for 45 min at room temperature with mixing. The Gα-subunits were then captured during a 3h room temperature incubation using a WGA-conjugated SPA beads (Perkin Elmer, 1 mg/reaction). The radioactivity counts of the bound GTP-γ-[^35^S] were determined by scintillation spectrophotometry (Wallac TriLux, Perkin Elmer). An EC_50_ value was determined by fitting the agonist response using a three-parameter fit model (GraphPad Prism 6.7)

## Results and discussion

### In silico comparative genomics of the M_4_ splice variant

**Fig 1:**
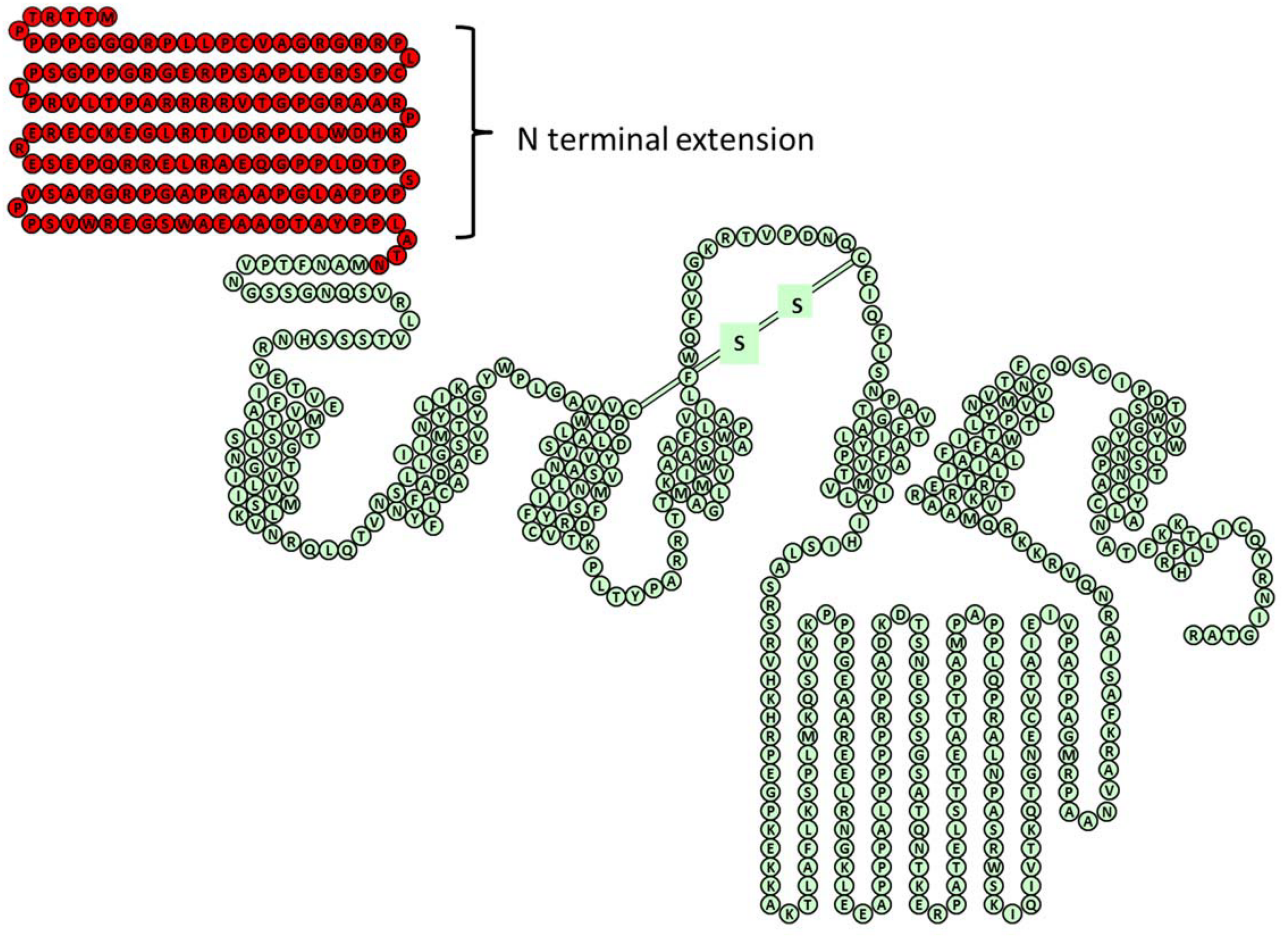
An amino acid sequence model of the muscarinic acetylcholine M_4_ receptor. Green represents the canonical sequence of the M_4_ receptor while red depicts the 155aa N-terminal extension.

A model of the predicted amino acid sequence for the M_4_L splice variant described in this manuscript can be found illustrated in Fig 1.

**Fig 2:**
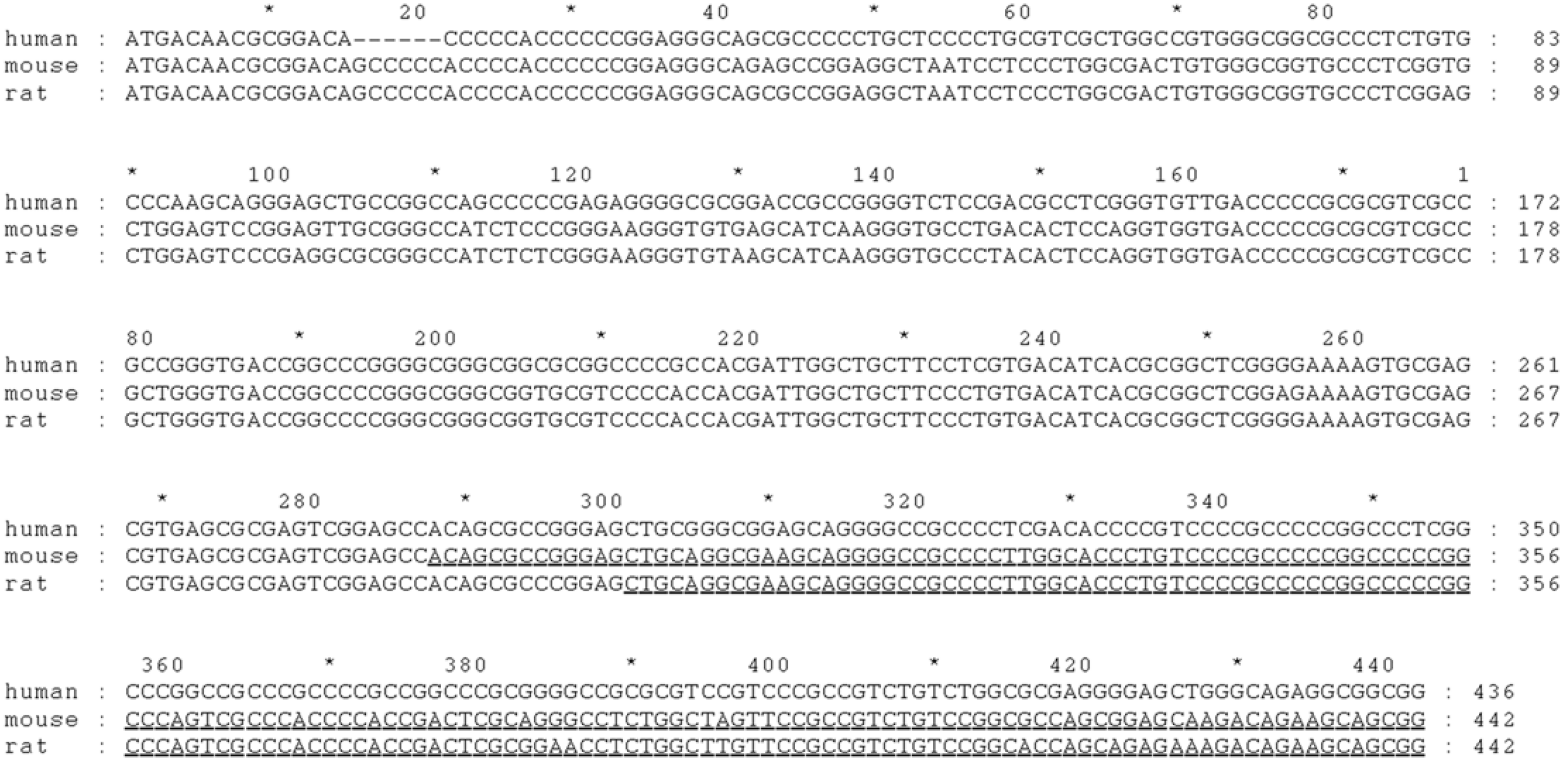
Upstream exon sequence alignments between human, mouse and rat. The underlined nucleotides in mouse and rat correspond to the EST sequences of BY250825.1, CJ145250.1, and DV213670.1.

BY250825.1 and CJ145250.1 are mouse ESTs that have two exons. DV213670.1 is a rat EST that also has two exons. These three ESTs were still “open” on the 5’ end relative to the open reading frame (ORF). The splice sites identified in the mouse and rat genomes for these three ESTs are conserved in the human genome. Comparative genomics suggests that an extension of the ORF is possible in human, mouse and rat up to a common ATG/methionine that could possibly extend the amino terminus by 155aa (from 31aa to 186aa for an amino terminus). The extended ORF of the three species is shown as an alignment in Fig 2.

The new Kozak consensus would be GtC cCC ATG and the new mRNA size (2 exons: new ATG to donor, acceptor to polyA signal) would be 3225 bp, and the new protein size would be 69.7 kDa.

### RNA-seq evidence for the existence of an M_4_ splice variant

**Fig 3:**
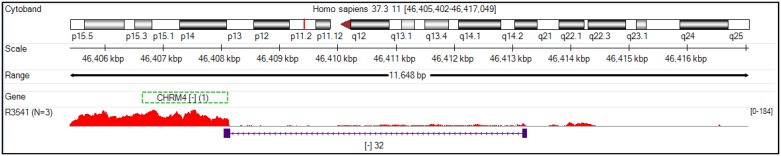
Junction reads through the M_4_L transcript from a human prefrontal cortex sample using OmicSoft Array suite.

The 211 human dorsolateral prefrontal cortex (DL-PFC) dataset confirmed the human splice junction of interest (11:46408136-^-46413169) that was originally identified from the rodent ESTs (Fig 3).

The upstream exon to create M_4_L was identified in 192 samples with 1426 total junction reads. Junctions Per Million reads Mapped (JPM) was calculated for each sample: JPM = (Junction Count) * 1000000 / (Total Reads Mapped). The average JPM is 0.08 and ranged from 0.86 to 0.

### PCR experimentation and sequence confirmation

**Fig 4:**
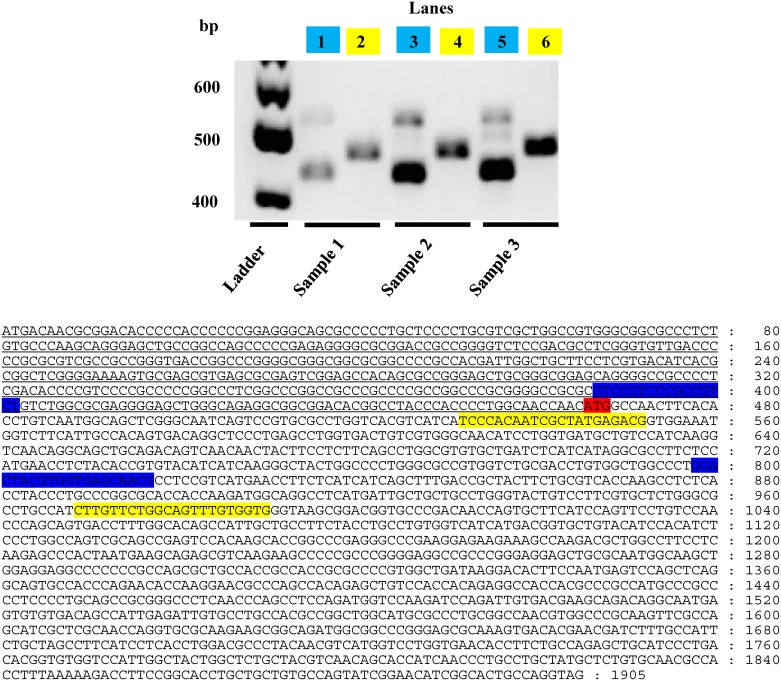
RT-PCR primer design and results.

Using the M_4_L transcript as our template (Fig 4), the following PCR primers were designed: The control primer set used to detect the canonical M_4_ transcript are highlighted in yellow while the junction primers designed to bridge between the short and long transcripts are highlighted in blue. Reverse transcription polymerase chain reaction (RT-PCR) reactions were performed on 3 human donor samples and their PCR products illustrated in Fig 4.

**Fig 5:**
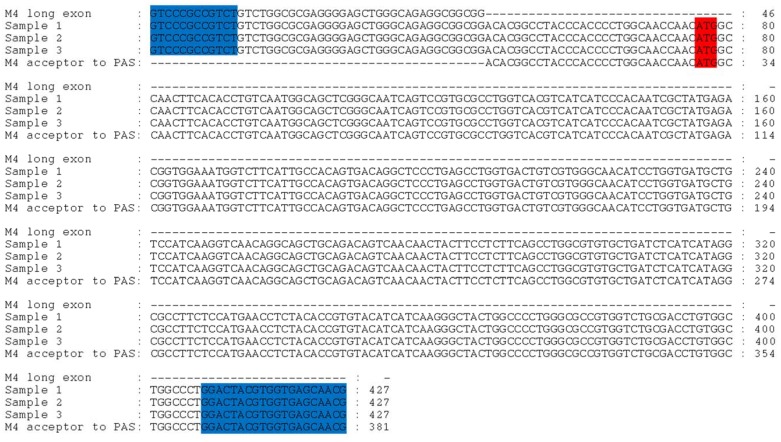
Sequence alignment of the 3 different donor samples used in this study. The sequences are aligned with the upstream M_4_L exon sequence (top sequence), has the CG-donor AC-acceptor sequence, and the canonical exon 1 sequence (bottom). The canonical start codon (ATG) is shown in red and blue represents the junction primers used in Fig 4.

Using splice junction-specific primers, RT-PCR experiments were performed to detect the junctions between the canonical M_4_ exon and the putative upstream exon within mature mRNAs from 3 different donor brains samples. Using the M_4_L transcript, the control primer set (shown in yellow) were used to detect the canonical M_4_ transcript while the junction primers designed to bridge between the short and long transcripts are highlighted in blue. The canonical start codon (ATG) is shown in red. RT-PCR reactions were performed on 3 human donor samples and their PCR products. All three samples were DL-PFC from control Caucasian female donors. Sample 1 (lanes 1&2) was from a 75 year old with a 50h PMI (postmortem interval), sample 2 (lanes 3&4) from an 83 year old with a 33.5h PMI and sample 3 (lanes 5&6) from a 49 year with a 36h PMI. Blue andyellow boxes represent the primer pairs mentioned above. All three samples were DL-PFC from control Caucasian female donors. Sample 1 (lanes 1&2) was from a 75 year old with a 50h PMI, sample 2 (lanes 3&4) from an 83 year old with a 33.5h PMI and sample 3 (lanes 5&6) from a 49 year with a 36h PMI. Using the control primer set (yellow) to detect the canonical M_4_ transcript in each of these samples, their PCR product can be seen in lanes 2, 4 and 6. The expected size of this primer combination was a 459bp product. The combination of the junction primers (blue) in lanes 1, 3 and 5 were designed to detect the junction of the canonical M_4_ exon with the putative upstream exon within a mature mRNA. The expected size of this primer combination was a 431bp product. Finally, the PCR products were cut and sequenced to confirm the existence of the junction for M_4_L. Fig 5 shows that the three human donor samples contain the putative upstream exon sequence; the sequences were aligned with the upstream M_4_L exon sequence (top sequence) and the canonical M_4_S exon 1 sequence (bottom).

### Expression of the M_4_S and M_4_L

**Fig 6:**
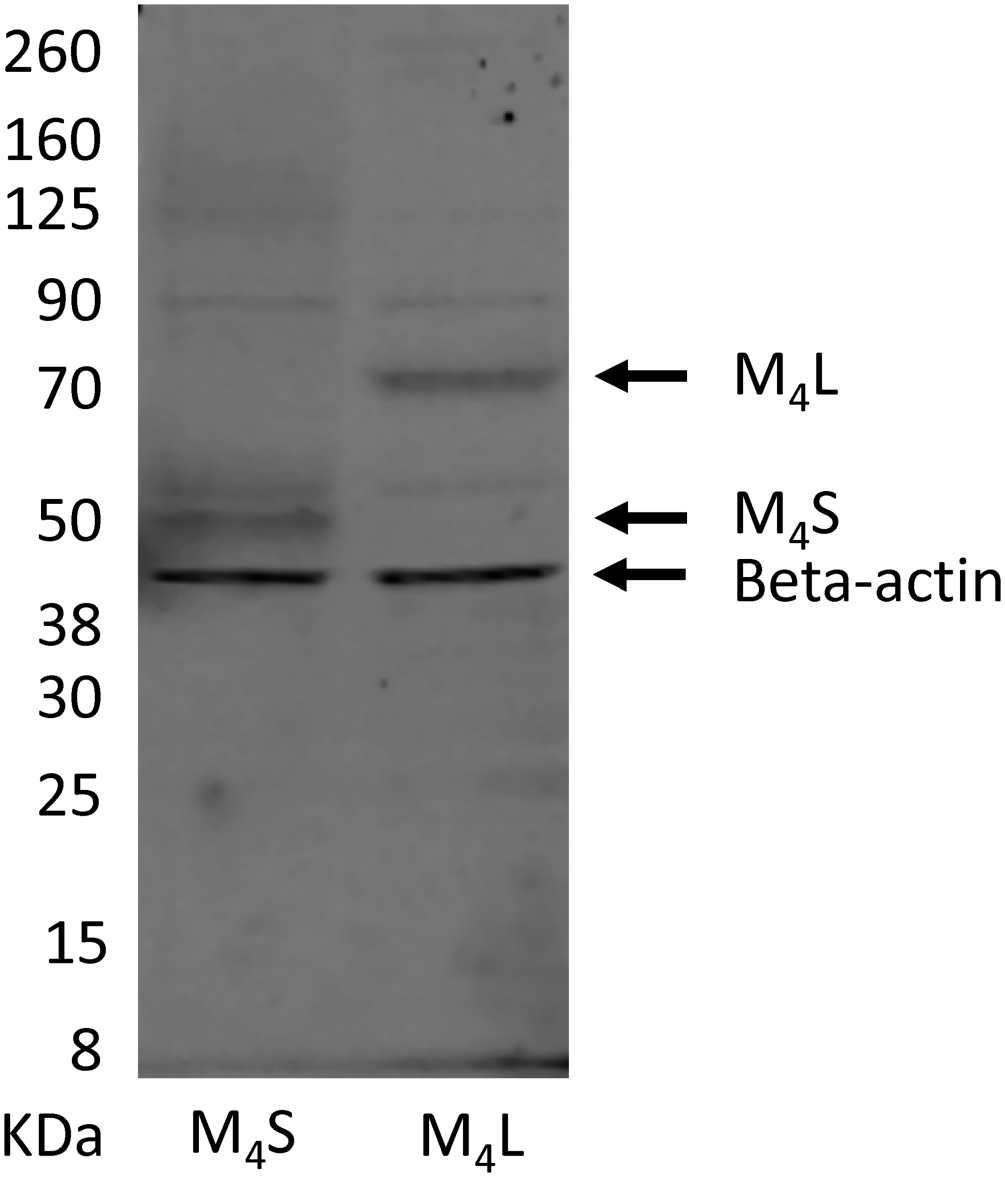
Western blot results for M_4_S and M_4_L transiently expressed in HEK293T. A representative Western blot on whole cell lysates from transient HEK293T cells expressing the M_4_S and M_4_L in the presence of an anti-myc antibody and a beta actin loading control monoclonal antibody.

HEK293T cells were transiently transfected with M_4_S and M_4_L constructs tagged at the N-terminus with myc. The M_4_S (theoretical molecular weight = 53kD) and M_4_L (theoretical molecular weight = 71kD) were detected in whole cell lysates using a commercially available myc antibody (Fig 6).

**Fig 7:**
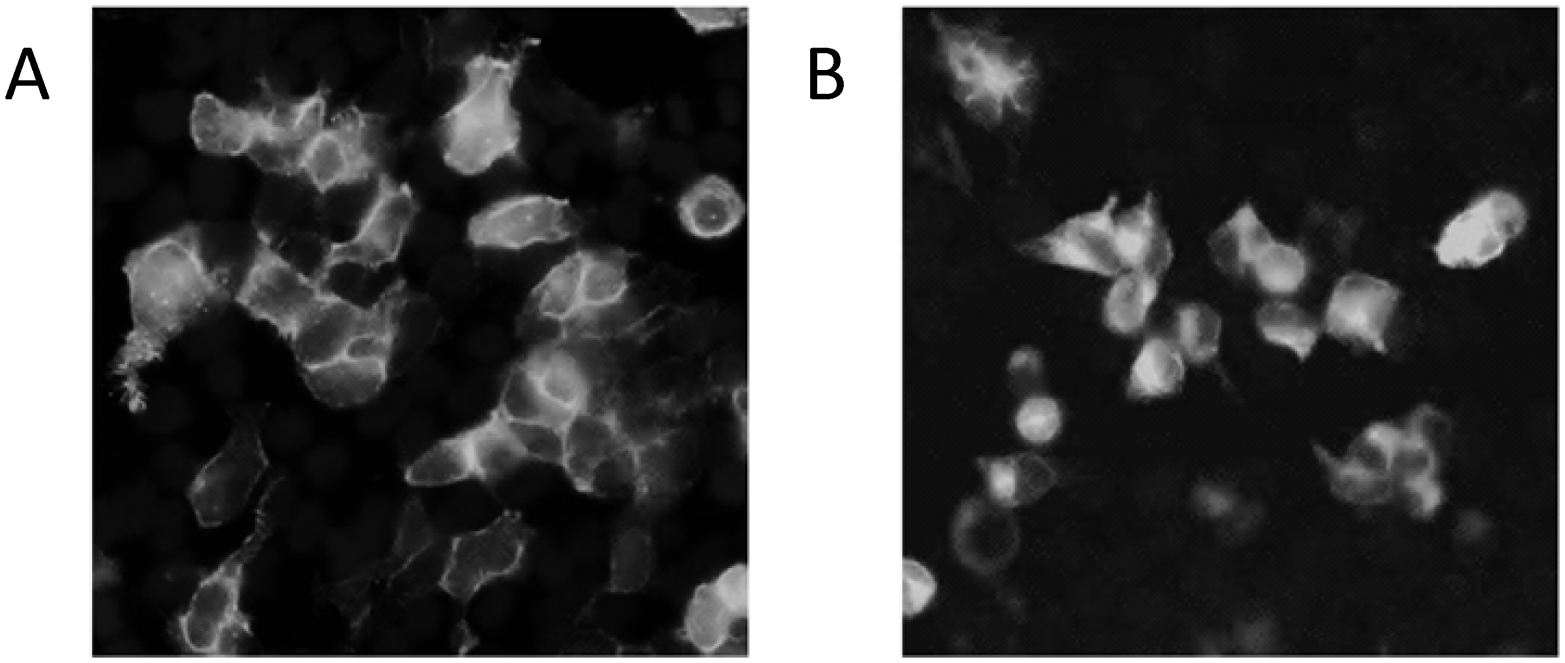
Immunoflorescence showing the distribution of M_4_S and M_4_L transiently transfected into HEK293T cells. A) Using an anti-myc antibody, image is representative of immunofluorescence of the M_4_S receptor transiently expressed in HEK293T cells. B) This image represents immunofluorescence of M_4_L transiently expressed in HEK293T cells.

Immuno-fluorescence was employed to investigate the cellular distribution of the M_4_S and M_4_L receptors in a HEK293T cell type stably expressing the constructs (Fig 7). Results indicate that M_4_S was mostly localized to the membrane (Fig 7A), conversely the M_4_L receptor appeared to localize mostly within the cytoplasm (Fig 7B).

Membranes assessed for saturation binding using [^3^H]-NMS showed that both M_4_S and M_4_L were expressed in HEK293T, however B_max_ values were much higher for M_4_S when cells were transfected with similar quantities of DNA. Conversely, the affinity (K_d_) for [^3^H]-NMS was nearly identical between M_4_S and M_4_L 0.13 ± 0.04 and 0.15 ± 0.02nM at 24μg DNA, respectively (Table 1). Overall, expression of both variants was sufficient for pharmacological characterization.

**Table 1:** Saturation binding with [^3^H]-NMS to membranes transiently expressing either M_4_S or M_4_L in HEK293T.

Receptor—μg DNA	B_max_ (fmol/mg protein)	K_d_ (nM)
M_4_S (48)	3120 ± 290	0.14 ± 0.03
M_4_L (48)	152 ± 13.8	0.12 ± 0.03
M_4_S (24)	1305 ± 38.5	0.13 ± 0.04
M_4_L (24)	745 ± .66.8	0.15 ± 0.02

Data shown are the result of 3 independent experiments performed in duplicate. The numerical values are expressed as the mean ± S.E.M. Using GraphPad unpaired t-test, no significant differences were found between the K_d_ values for both receptors. However, B_max_ values were statistically significant between M_4_S and M_4_L at 24 (P = 0.002) and 48 (P = 0.0005) μg DNA.

### Comparison between the M_4_S and M_4_L orthosteric site assessed by GTP-γ-[^35^S] binding

**Fig 8:**
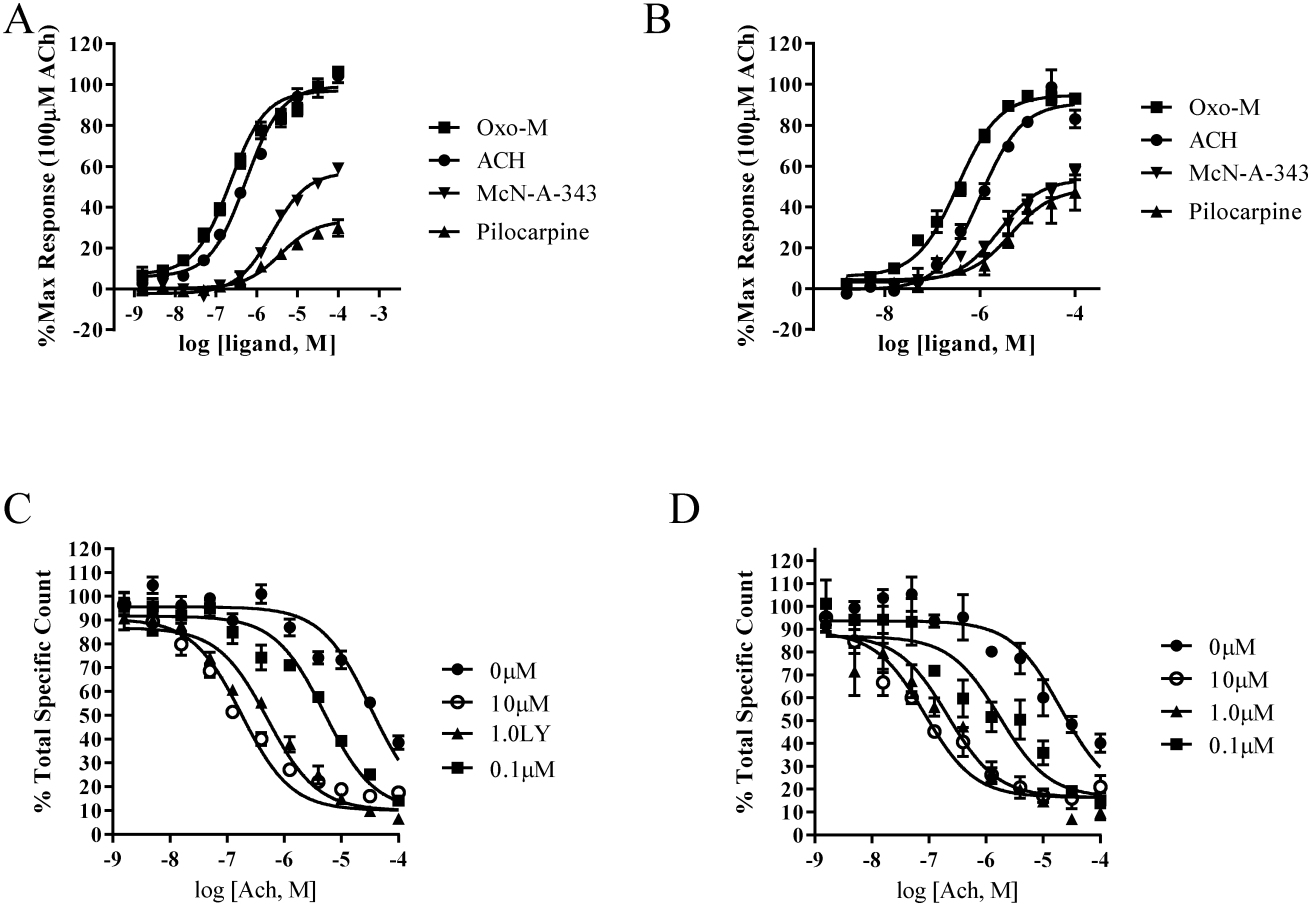
Functional comparison of the orthosteric and allosteric binding sites between the M_4_S and M_4_L receptors. A) Represents the orthosteric response of the M_4_S to known muscarinic agonists: acetylcholine (closed circle), oxotremorine-M (square), pilocarpine (triangle) and McN-A-343 (inverted triangle). B) Summary of the M_4_L response for the same 4 muscarinic agonists. C) Graph represents the allosteric response of the M_4_S receptor to ACH in the presence of various concentrations of the M_4_-PAM, LY2033298. D) Allosteric response of M_4_L to ACH in the presence of zero (closed circle), 0,1μM (square), 1.0μM (triangle) or 10μM (open circle) LY2033298. Data shown are the result of 3 independent experiments performed in duplicate.

Fig 8A and 8B summarizes the functional concentration response curves for acetylcholine, oxotremorine-M (Oxo-M), McN-A-343 and pilocarpine stimulated GTPγ-[^35^S] binding in cells transiently expressing M_4_S (Fig 8A) and M_4_L (Fig 8B).

Both acetylcholine (circle) and oxo-M (square) displayed full agonist properties at M_4_S and M_4_L receptors. In addition, the EC_50_ values for acetylcholine and oxo-M were very similar at both receptors. Acetylcholine had an EC_50_ = 619±47.9nM and 714±231nM for the M_4_S and M_4_L, respectively. Likewise, oxotremorine-M EC_50_ at the M_4_S was 310±120nM and 270±118nM at the M_4_L variant. Both pilocarpine (triangle) and McN-A-343 (inverted triangle) showed partial agonist activity at the M_4_L receptor consistent with their known pharmacology at the canonical M_4_ receptor. Similar results were found with commercially available membranes containing the canonical M_4_S receptor.

### Comparison between the M_4_S and M_4_L allosteric site assessed by [^3^H]-NMS competitive binding

A series of [^3^H]-NMS binding experiments were performed to assess whether differences in the allosteric binding sites existed between the M_4_S and M_4_L receptor variants. The displacement of [^3^H]-NMS by oxo-M was very similar between the M_4_S and M_4_L with K_i_ log values of 5.25±0.10nM and 6.00±0.26nM respectively (Fig 8C and 8D). Competitive binding experiments were then used to assess the function of a common muscarinic allosteric binding site located in the outer extracellular vestibule of muscarinic receptors [19, 20]. Specifically, the potentiation of oxo-M was tested in the presence of varying concentrations of LY2033298, an M4 positive allosteric modulator (M_4_-PAM). In the presence of 10μM LY2033298 oxo-M was potentiated 100-fold at both the M_4_S and M_4_L receptors similar to previous reports [1]. The M_4_S receptor had a K_i_ log value of 7.52 ± 0.06 which was similar to the M_4_L which had a K_i_ log value of 8.05±0.15 at this concentration of an M_4_-PAM.

## Conclusions

Northern/Western data from the literature support this possibility that the muscarinic receptor family contains splice variants [15, 16]. In fact, a common alternative splice variant was identified in mouse and rat. BY250825.1 and CJ145250.1 are mouse ESTs that show an upstream exon in addition to the exon that contains the currently understood, complete open reading frame. DV213670.1 is a rat EST that covers the comparable two exons. In the open reading frame analysis, there are no in-frame upstream stop codons and the chosen Kozak consensus sequence is not very strong. The splice donor and acceptor sites identified in the mouse and rat genomes for these three ESTs are conserved in the human genome. Comparative genomics reveals that an open reading frame is possible in human, mouse and rat up to a common ATG/methionine that could possibly extend the amino terminus by 155aa. The new Kozak consensus would be GTC CCC ATG a, the new mRNA size (2 exons: new ATG to donor, acceptor to polyA signal) would be 3225 bp, and the new protein size would be 69.7 kDa. Preliminary RT-PCR experiments were performed to detect the human upstream exon and the connectivity of the two exons within a mature mRNA. The expected size for this splice variant was detected, as well as others. Taqman identified that this particular splice variant is also expressed in brain, testes and thymus.

Additional experiments were performed to identify, express and pharmacologically characterize this splice variant at the protein level. Saturation radioligand binding with [^3^H]-NMS (N-methyl-scopolamine) from HEK293T membranes transiently expressing M_4_S and M_4_L revealed that new splice variant can be expressed. However, to determine that the M_4_L was truly expressed and not just M_4_S that had been post-translational modified, the same membranes were subjected to Western blot analysis. Both M_4_S and M_4_L were tagged at the N-terminus with myc so that they can be identified with a commercially available anti-myc antibody. Western blot analysis detected in whole cell lysates, bands of 53kD and 71kD, the theoretical molecular weights for M_4_S and M4L, respectively. Comparative pharmacological characterization between the M_4_L and M_4_S receptors revealed that both the orthosteric and allosteric binding sites for both receptors were very similar despite the addition of an N-terminal extension.

It has long been known that alternative splicing was an important post-transcriptional process by which diverse transcripts can be generated from one mRNA precursor and first proposed in 1978 [21]. Alternative splicing has been recognized as a major source of transcriptome and proteome diversity in GPCRs as well [6]. GPCR receptor splice variants could result in a multitude of pharmacological behaviors. Genetic variation in G-protein coupled receptors has been shown to be associated with a wide spectrum of disease phenotypes and predispositions that are of special significance because they are amenable targets for therapeutic agents [22]. The N-terminus has been shown to be the most variable element in GPCRs, ranging from seven to approximately 5900 residues [23]. One important function of the N-terminus is to stabilize the first transmembrane helix to ensure the correct receptor structure. Several alternative splicing of exons encoding the N-terminal domain have been reported and have been shown to display altered ligand affinity as well as differential activation by endogenous ligands. For example, N-terminal splice variants of the type I PACAP receptor has been shown to have both altered binding and cAMP function with the PAC1 ligand. This leads to significant differences in the affinities and selectivity towards PACAP38, PACAP27 and VIP in the tested HEK293 cell model [24].

Here we describe a new splice variant for the muscarinic M_4_ receptor. Using a combination of bioinformatics, molecular and cellular biological techniques we have evidence for an N-terminal extension of the M_4_ receptor that results in a unique receptor isoform that can be exogenously expressed. In this communication, binding to the orthosteric and allosteric binding pockets for both the M_4_S and M_4_L receptors were not compromised by altering the N-terminus, but remain very similar to each other. Recent crystallization of the active state of the muscarinic M_2_ receptor with both allosteric and orthosteric sites occupied provided detailed information of both binding pockets [19]. Since LY2119620, the M_4_ allosteric ligand docked into the active state M_2_ crystal also modulates the M_4_ receptor, their structural and binding are likely similarity [25, 26]. Therefore, the extension of the N-terminal domain most likely does not provide unique orthosteric or allosteric binding regulation, but that does not rule out that splice variants don’t provide an opportunity to redefine the physiology and pharmacology of known muscarinic receptor family.

The most notably different between the M_4_S and M_4_L was expression when expressed in HEK293T cells. The N-terminal extension isoform appears to have an effect on receptor expression at the membrane. Using immuno-fluorescent, the M_4_S receptor appeared to be mostly expressed on the cell membrane (Fig 7A) while M_4_L expression appears more cytosolic (Fig 7B). This pattern was irrespective of M_4_L expression. This pattern was very reminiscent to the V2a receptor. The V2a receptor did not move to the plasma membrane, but was retained in the ER—Golgi compartment [27]. Our results are very similar to the somatostatin receptor sst5, which has two truncated isoforms named sst5TMD5 and sst5TMD4. In contrast with the predominant plasma membrane localization of full-length sst5, both sst5TMD5 and sst5TMD4 show a preferential intracellular localization [28]. Another splice variants, C1a receptor [29] has also been shown to result in poor receptor expression at the plasma membrane so our findings are not unprecedented. Although the biological implication of the M_4_L localization needs to be further explored, mis-localization may not be abnormal since as much 50% of newly synthesized proteins are ER retained [30].
